# A Comparison of Ki67, Syndecan-1 (CD138), and Molecular RANK, RANKL, and OPG Triad Expression in Odontogenic Keratocyts, Unicystic Ameloblastoma, and Dentigerous Cysts

**DOI:** 10.1155/2018/7048531

**Published:** 2018-07-29

**Authors:** Luisana Brito-Mendoza, Ronell Bologna-Molina, María Esther Irigoyen-Camacho, Guillermo Martinez, Celeste Sánchez-Romero, Adalberto Mosqueda-Taylor

**Affiliations:** ^1^Oral Medicine and Pathology Postgraduate Program, Health Care Department, Universidad Autónoma Metropolitana Xochimilco, Mexico City, Mexico; ^2^Molecular Pathology, Faculty of Dentistry, Universidad de la República, Montevideo, Uruguay; ^3^Oral Pathology, Department of Oral Diagnosis, Piracicaba Dental School, University of Campinas, Piracicaba, Brazil

## Abstract

**Background and Objective:**

Reduced expression of syndecan-1 (CD138), increased proliferation index, and modifications in the expression of the molecular RANK/RANKL/OPG triad are related to an intensified potential of aggressiveness and invasion of diverse tumors and cysts. The aim was to compare the expression of Ki-67, CD138, and the molecular triad RANK, RANKL, and OPG in odontogenic keratocysts (OKC), unicystic ameloblastomas (UA), and dentigerous cysts (DC).

**Methods:**

Immunohistochemistry for Ki-67, CD138, RANK, RANKL, and OPG was performed in 58 odontogenic cystic lesions (22 OKC, 17 DC, and 19 UA).

**Results:**

A higher expression of Ki-67 was identified in OKC as compared to UA (*p* < 0.0001). UA exhibited a greater loss of CD138 expression versus OKCs (*p* > 0.0034). RANKL was expressed higher in the epithelium (*p* = 0.0002) and in the stroma (*p* = 0.0004) of UA. DC had a lower expression of these markers.

**Conclusion:**

Higher RANKL expression together with the reduction on CD138 expression in UA could be linked to a greater invasive and destructive potential, while the increased proliferation rate observed in OKC could be related to its continuous intrabony growth. The expansion of DC does not seem to be related to such factors, justifying the different therapeutic approaches proposed for each of these entities.

## 1. Introduction

Cystic lesions of odontogenic origin are responsible for a large number of extensive surgeries, which can be due, at least in part, to the progressive and infiltrative growth of some of them, as well as to their ability to induce bone resorption and to increase the risks of a pathological fracture, secondary infections, and diverse functional and esthetic disorders of the maxillofacial region.

At the epithelial lining of such lesions, there are proteins responsible for maintaining the intracellular adhesion, as well as their adhesion with their extracellular matrix. Among these, CD138 (syndecan-1), a protein encoded by the SDC1 gene, is in charge of mediating the adhesion between cells and between the cells and the extracellular matrix, participating also in cell signaling and in the cytoskeleton arrangement, and acts as an integral part of the membrane proteins which also participate in cell proliferation, cell migration, and cell-extracellular matrix interactions. Some studies have shown that a reduction in its expression might be related with the ability of epithelial invasion to the capsule and adjacent structures [1, 2].

Ki-67 is a nuclear protein considered as a cell proliferation marker, since it is present during the active stages of the cell cycle, and its expression is usually related to the biological behavior of cells (growth speed and proliferative potential) [3].

Among the proposed expansion mechanisms of cystic lesions of the odontogenic origin, those related to the induction of bone destruction have a leading role. Such bone destruction is found mostly regulated by a molecular RANK, RANKL, and OPG triad, a group of glycoproteins related to bone resorption. An unbalance of these factors is observed in patients with osteoporosis, osteopetrosis, rheumatoid arthritis, periodontal diseases, and even in altered tooth eruption. This triad is formed by molecules that can regulate bone homeostasis and particularly osteoclast maturity [4]. The receptor activator that triggers the *κβ* nuclear factor (RANK) belongs to the family of the tumor necrosis factor, and this is triggered by RANK ligand (RANKL), a cytokine similar to TNF. RANK is located at the degraded bone surface and expressed in the surface of osteoclasts. Osteoprotegerin (OPG) is a soluble receptor that disrupts osteoclast activation by binding directly to RANK; therefore, osteoclast maturity and bone homeostasis are given by balanced RANK-RANKL and OPG levels [4, 5].

Unicystic ameloblastoma (UA) and odontogenic keratocyst (OKC) are benign but locally invasive lesions. They are the two cystic entities with the highest potential of destroying the maxillofacial skeleton. Both are characterized by a structure similar to other nonneoplastic cystic lesions and also share clinical and radiological features with many of them. Very often UA and OKC mimic a dentigerous cyst (DC), and consequently, it is necessary to include them in the differential diagnosis [6–9]. To understand the similarities and differences among them that could be useful for diagnosis and therapeutic purposes, it is important to study the cellular and extracellular elements that take part on the physiopathological mechanisms implicated in their origin and growth; therefore, the aim of this study was to evaluate and compare Ki67, CD138, and the molecular RANK, RANKL, and OPG triad expression on the lining epithelium and stroma of UA, OKC, and DC.

## 2. Material and Methods

Cases from the archives of the Oral Pathology Laboratory from the Universidad Autónoma Metropolitana Xochimilco and from a private diagnosis center of oral and maxillofacial pathology located in Mexico City were included in this study. A total of 58 cases were included in this study (22 OKC, 19 UA, and 17 DC). The inclusion criteria were diagnoses which fulfilled the histological parameters described in the most recent classification of odontogenic cysts and tumors of the WHO [10]. Only those lesions that did not exhibit inflammation at the interface of the fibrous capsule and the lining epithelium were included. OKCs linked to nevoid basal cell carcinoma syndrome were excluded.

### 2.1. Immunohistochemistry Technique

2 *μ*m-thick sections were mounted on plates treated with poly-L-lysine. The slices were deparaffinized in a stove at 45°C for 30 minutes and, afterwards, placed in xylol for 5 minutes. Slices were hydrated in a train in the following descending concentration order: absolute alcohol, 90, 70, and 50%, and slices were rinsed with distilled water. In order to unmask epitopes, antigen retrieval with sodium citrate 10 mM (pH 6.2) with a pressure cooker in microwave at a maximum pressure (750 W) for 5 minutes was used. It was allowed to cool at room temperature, and it was then rinsed with distilled water. Endogenous peroxidases were blocked with hydrogen peroxide 0.9% and washed with distilled water and phosphate-buffered saline (PBS) pH 7.4. Slides were mounted with coverslips and were placed on supporting frames in order to perform immunohistochemistry. Monoclonal primary antibodies were incubated at room temperature for 45 minutes. The following antibodies were used: anti-OPG (clone ab73400 Abcam laboratories), anti-RANK (clone 64C1385 Abcam laboratories), anti-RANKL (clone 12A668 Abcam laboratories), anti-Ki-67 (clone L26 Dako laboratories), and anti-CD138 (clone B-A38 Biocare laboratories). Afterwards, slices were incubated with the second biotinylated anti-mouse/anti-rabbit antibody and streptavidin/peroxidase complex (LSA-B (labeled streptavidin-biotin), Dako Corporation, Carpinteria, CA, USA) for 30 minutes each. Reaction products were observed with 3,3′-diaminobenzidine-H_2_O_2_ (Dako Corporation, Carpinteria, CA, USA) substrate. The sections were counterstained with Mayer's hematoxylin.

A reviewer evaluated the immunohistochemical reaction of the lesions, after being standardized by a peer pathologist and a *k* = 0.75 kappa was obtained.

Five fields for each case were selected. It was considered as positive immunostain for Ki-67 in cystic lining epithelium when all those epithelial cells exhibited positive stain at the nucleus. For CD138, it was considered as positive immunostain when all those cystic lining epithelium cells exhibited stain at cell membrane, while for RANK, RANKL, and OPG, it was considered as positive immunostain when the cystic lining epithelium cells and the stroma cells exhibited cytoplasm stain. The immunoreactivities of nuclear markers (only Ki-67) were assessed via a semiquantitative method described by Bologna-Molina et al. [3]. In brief, a 400x microphotograph per field (five fields per case) was taken. The image was transferred to a grid in order to make the counting easier. The number of Ki-67-positive cells was divided by the total of cells present at the epithelial lining and multiplied by 100, obtaining the cell proliferation index (label index). Then, five fields per each case were averaged.

In the analysis of CD138, a semiquantitative evaluation was made of all the epithelial membranes obtaining a percentage; it was considered as 100% immunostaining when all the epithelial membranes of the cyst lining were positive. For RANK, RANKL, and OPG immunostaining, only the number of positive cells located at the cystic lining epithelium and stromal cells were counted and added, obtaining finally a total mean of positive cells, both in the epithelium and the stroma of each lesions.

### 2.2. Statistical Analysis

Means, standard deviations (SD), and medians were obtained to characterize the presence of bone reabsorption markers (RANK, RANKL, and OPG) and for cell adhesion and proliferation markers (Ki-67 and CD138) in the studied lesions. To compare categorical data, a chi-squared test was applied. Nonparametric tests (Kruskal-Wallis) were used to identify differences between the medians of immunoexpression values among the three types of lesions. To compare the immunoexpression of the markers, the Tukey-Kramer method (honestly significant difference) was utilized. This allows testing of these hypotheses, keeping type I (*α* = 0.05) error. Additionally, Spearman's Rho (*ρ*) was calculated to study the correlation between the numbers of positive cells (Ki-67, CD138, RANK, RANKL, and OPG) from the different types of lesions. Hypothesis tests were conducted for *p* < 0.05. StataCorp V. 10 software was used.

## 3. Results

**Figure 1:**
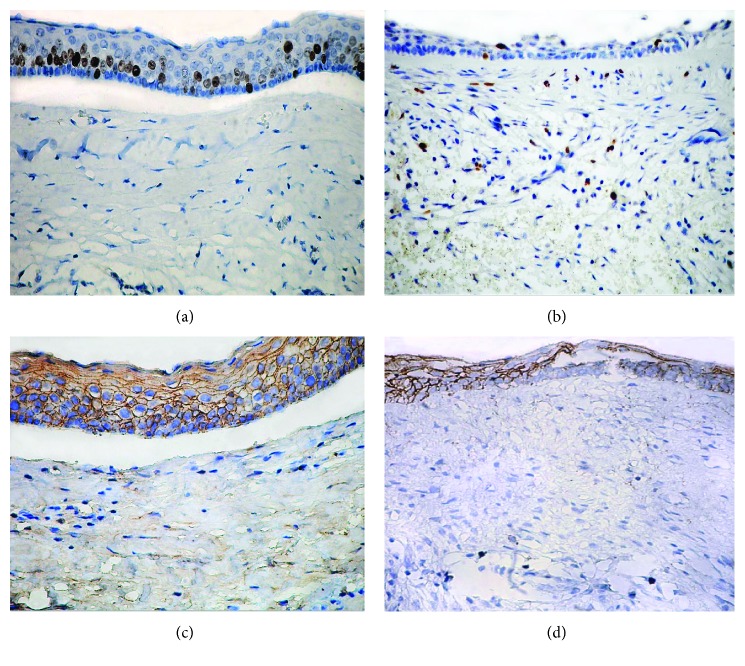
Comparison between Ki67 expression in odontogenic keratocyst (a) and unicystic ameloblastoma (b) versus CD138 expression in OKC and UA (c, d). Immunoperoxidase (40x).

Regarding Ki-67 expression, the mean of proliferative index observed in OKC was 17.71 (±SD 6.29) with a median of 17. This was the lesion with the highest proliferative index. Significant differences were observed among the three lesions (*p* < 0.0001). Additionally, significant differences on Ki67 expression were detected between OKC and UA and between OKC and DC (*p* < 0.0001). Representative pictures of the immunohistochemical expression of Ki-67 and CD138 are shown in Figure 1.

**Figure 2:**
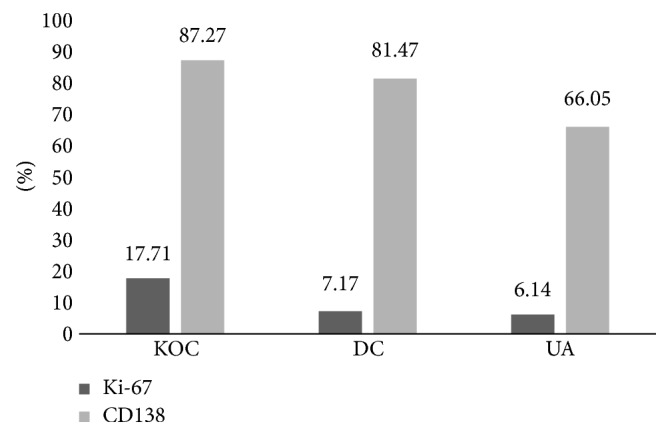
Percentage of positive cells that expressed Ki-67 and CD138 in the epithelia of odontogenic keratocyst (OKC), dentigerous cyst (DC), and unicystic ameloblastoma (UA).

The CD138 expression OKC had the greater number of positive cells present in the epithelium (mean: 87.27 ± 16.52) (*p* = 0.0093). Likewise, significant differences were found on CD138 expression when comparing OKC and UA (*p* < 0.0034).  Figure 2 shows mean Ki67 and mean CD138 values on the three lesions studied, observing significant differences among the three lesions (*p* < 0.05). It is interesting to note that a positive correlation between cell proliferation index (Ki-67) and CD138 expression was also observed; the lesser the CD138 expression, the lesser the cell proliferation index mean is (Figure 2).

**Figure 3:**
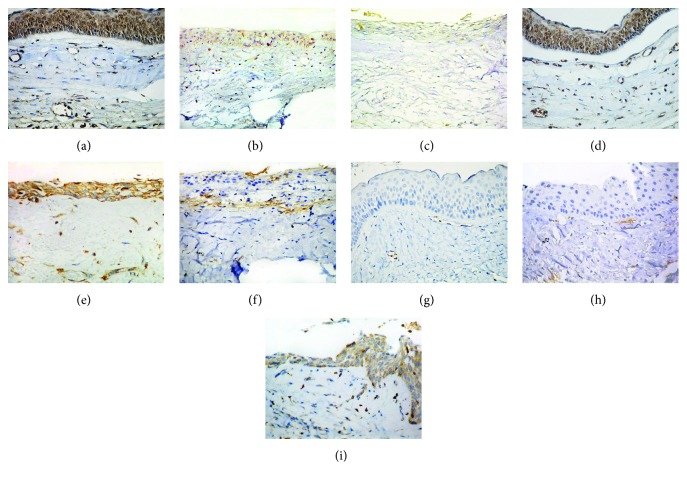
Comparison between OPG, RANK, and RANKL expression in the epithelium of OKC (a, d, and g), DC (b, e, and h), and UA (c, f, and i). Immunoperoxidase (40x).

No statistically significant differences were observed in the mean of positive cells (absolute number) for RANK among the three groups of lesions studied with this marker (*p* > 0.05) (Table 1). OKC's stroma had the higher mean of positive cells. A significant difference (*p* = 0.0206) was observed in RANK expression in the stroma of the three lesions studied. Additionally, a statistically significant difference was detected between OKC and DC (*p* = 0.0036). Representative pictures of the immunohistochemical expression of RANK, RANKL, and OPG are shown in Figure 3.

UA was the lesion with the highest mean of RANKL-positive cells in the lining epithelium and stroma. We found statistically significant evidence in the comparison of the expression of RANKL in the epithelium and stroma (Table 1).

Differences were found when comparing OPG expression at the lining epithelium of each of the lesions between. Additionally, a significant difference was found when comparing OPG expression in the stroma of the three lesions included in this study. A positive correlation (Spearman) was observed between RANKL and OPG expression at the epithelium (*ρ* = 0.34, *p* = 0.0033) and in the stroma of the UA (*ρ* = 0.35, *p* = 0.0264).

## 4. Discussion

This study compares the expression of different immunohistochemical markers aiming at analyzing the cell proliferation index (Ki67), the expression of cell adhesion molecules (CD138), and factors that regulate bone reabsorption (RANK, RANKL, and OPG) in three groups of cystic lesions of the maxillofacial region free of inflammation. Even though some of the possible growth and expansion mechanisms of cystic lesions of odontogenic origin have been previously studied [11–13], the specific function of each of the cellular and extracellular elements implicated has not been completely defined. Therefore, we consider that studies like this are necessary to better evaluate this group of lesions that seem to be similar from a clinical point of view but differ in their histopathology and biological behavior.

It is well known that OKC exhibits a more aggressive behavior than the other odontogenic cysts, which might be due, among other reasons, to its higher cell proliferation index [14–19]. In agreement with this, in our study, we observed that OKC was the lesion with the highest cell proliferation index, exhibiting a significant difference when compared to DC and UA. These results highlight the existing differences among these entities and support the concept that OKC behaves in a similar way to locally aggressive jaw tumors, with a high proliferative index. This fact would explain to a great extent its persistent growth but with less bone-resorbing activity and invasive ability, demonstrated by its higher expression of CD138 compared to what is seen in UA. This could explain why OKC reaches a very large size before being discovered and only some cases demonstrate epithelial invasion to the fibrous capsule.

As one of the main functions of CD138 is to preserve cell-cell and cell-extracellular matrix adhesion, we consider that it is important to compare the high expression and the elevated rate of cell proliferation mediated by Ki-67 found in the epithelial lining of OKC with the low expression of both immunomarkers recorded in UAs. We concluded that these differences may be related to a loss of cell adhesion of the UAs, which might explain the frequently detected epithelial invasion it produces into the capsule and adjacent bone, leading to “mural” proliferation [8, 20]. In other words, the high proliferation index is not a sufficient thesis to explain the biological behavior of a cystic lesion in terms of aggressiveness, infiltration, or bone destruction, but it can help to explain its persistent growth and expansive ability. On the other hand, the loss of expression of CD138, as has been evidenced in some benign locally infiltrating lesions, such as the ameloblastomas and in malignant epithelial neoplasms, can be considered as an important sign that may be related to a higher infiltration ability of a specific lesion. In addition, the positive correlation of such factors together (Ki67 and CD138) provides elements to explain the different biological behaviors observed among the lesions included in this study [1].

During the last years, some studies have been published which correlate the presence of these two proteins with the behavior of some odontogenic tumors. Our results are supported by a previous study [1] that conducted a comparative study of CD138 and Ki-67 expression in different types of ameloblastomas, identifying that the solid type has the lower percentage of expression of CD138 (40.2% versus 49.7% found in UA), and they concluded that this might be one of the reasons why solid ameloblastomas (SA) tend to be more aggressive than its unicystic variant [21]. Bologna-Molina et al. [1] also compared CD138 expression in ameloblastic carcinomas, peripheral ameloblastomas, and desmoplastic ameloblastomas, and they found the greater loss of this component in ameloblastic carcinoma. This fact might be related with malignant transformation of conventional intrabony ameloblastomas versus lesions with less aggressive behaviors, such as the desmoplastic and peripheral types of ameloblastoma [1].

Recently, Al-Otaibi et al. [2] conducted a comparative analysis of CD138 expression in solid ameloblastomas, UA, OKC, and DC. They found significant differences among the three groups, as UAs exhibited a reduction on the expression as compared to OKC and DC, which expressed it in more than 90% and 100%, respectively. In our cases, immunoexpression in DC was observed in 81.47%. This might be explained by differences in the conditions of the tissues examined, since such authors do not specify if the DCs studied exhibited inflammatory reaction or squamous metaplasia, which might explain the difference on CD138 expression as compared to our findings, which were registered solely in cases with no inflammation.

In addition to the study of the elements involved in epithelial proliferation and adhesion, we performed an immunohistochemical analysis of the main elements involved in the bone resorption mechanism, specifically the RANK-RANKL-OPG triad. In the previous study, de Moraes et al. [22] showed that the epithelial lining of cysts exhibits immunostaining for RANK, RANKL, and OPG in cells of the basal and suprabasal layers. These authors state that the expression in the odontogenic epithelial cells suggests that the odontogenic epithelium may actually induce and initiate the resorption process, perhaps through synthesizing and secreting RANKL and OPG.

da Silva et al. compared the expression of this triad in the epithelial lining and in the stroma of OKC, UA, SA, and DC and did not observe differences in the expression of RANKL and OPG at the epithelial level [23]. A similar result was identified in the present study, where no differences in RANK expression were identified in the lining epithelium of the three lesions included, although increased expression of RANK, RANKL, and OPG was found in the epithelium as compared to the stroma. On the other hand, OPG expression was lower in both epithelium and stroma as compared to RANKL or RANK expression, which differs from that found by da Silva et al. [23]. This difference could be due to the characteristics of the studied samples, since the study by da Silva et al. [23] included lesions with moderate and even abundant inflammatory infiltrate, as well as some OKCs associated with the nevoid basal cell carcinoma syndrome. In addition, in our study, UA was the entity that significantly showed a higher number of RANKL-positive cells and a lower number of positive cells for OPG, a finding that is in agreement with what was found by da Silva et al. [23].

In general terms, the moderate proliferation index detected on the UA suggests that although this neoplasm grows by the expansion of its epithelial lining, the lower CD138 expression observed in comparison to OKC and DC could explain its higher invasive and destructive ability that allows the neoplastic cells of UA to extend into adjacent tissues [2]. This seems to be reinforced by its higher RANKL expression, which is linked to osteoclastogenesis activation and favors the destruction of cortical walls, an event only observed in late stages or very advanced stages in OKC growth and other less aggressive lesions [23].

OKC exhibited a cell proliferation index higher than UA and DC. This finding suggests that OKC grows basically as a result of cell proliferation of its epithelial lining, having less participation of the mechanisms possibly associated to intercellular separation and invasion to the capsule. Likewise, the expansion of OKC is favored by the expression in the epithelium of factors which stimulate osteoclast maturation through the TNF-*α* expression [24].

In conclusion, a higher RANKL expression together with the reduction on CD138 expression in UA could suggest a greater invasive and destructive biological behavior, while the increased proliferation rate observed in OKC could be related to its continuous intrabony growth. The expansion of DC does not seem to be related to such factors.

## Figures and Tables

**Table 1:** Positive cell mean for RANK, RANKL, and OPG in odontogenic keratocyst, dentigerous cyst, and unicystic ameloblastoma.

Type of lesion (number of cases)	RANK		RANKL		OPG	
	Mean	*p*	Mean	*p*	Mean	*p*
Lining epithelium						
OKC (22)	180.95	0.3879^a^	43.59	0.4153^g^	117.90	0.0121^m^
DC (17)	249.47	0.7693^b^	26.52	0.0317^h^	11.47	0.0211^n^
UA (19)	212.47	0.8053^c^	62.47	0.3205^i^	6.89	0.9928^o^
Stroma						
OKC (22)	101.38	0.0036^d^	34.27	0.0004^j^	54.09	0.0018^p^
DC (17)	169.94	0.3072^e^	27.11	0.5292^k^	14.35	0.0196^q^
UA (19)	139.00	0.1414^f^	60.73	0.0001^l^	4.00	0.7629^r^

*χ* ^2^ = 1.8886 (RANK epithelium), *χ*^2^ = 7.7653 (RANK stroma), *χ*^2^ = 13.3295 (RANKL epithelium), *χ*^2^ = 15.9150 (RANKL stroma), *χ*^2^ = 4.2004 (OPG epithelium), *χ*^2^ = 12.24 (OPG stroma). OKC: odontogenic keratocyst; DC: dentigerous cyst; and UA: unicystic ameloblastoma. Mean differences test by pair of lesions (Tukey): ^a^OKC versus DC, ^b^DC versus UA, ^c^UA versus OKC, ^d^OKC versus DC, ^e^DC versus UA, ^f^UA versus OKC, ^g^OKC versus DC, ^h^DC versus UA, ^i^UA versus OKC, ^j^OKC versus UA, ^k^DC versus OKC, ^l^UA versus DC, ^m^OKC versus UA, ^n^DC versus OKC, ^o^UA versus DC, ^p^OKC versus UA, ^q^DC versus OKC, ^r^UA versus DC.

## Data Availability

The immunohistochemical quantification data used to support the findings of this study are included within the article. Any other additional data are available from the corresponding author upon request.
